# Physiological, technical, and time-motion responses according to small-sided game formats and pitch size variations in adolescent male soccer players: effects of biological maturity status

**DOI:** 10.5114/biolsport.2025.146788

**Published:** 2025-01-16

**Authors:** Bilel Cherni, Hamza Marzouki, Okba Selmi, Yung-Sheng Chen, Anissa Bouassida, Karim Chamari

**Affiliations:** 1 High Institute of Sport and Physical Education of Ksar-Said, University of Manouba, Tunisia; 2 Research Unit: Sport Sciences, Health and Movement, University of Jendouba, Kef, Tunisia; 3 High Institute of Sport and Physical Education of Kef, University of Jendouba, Tunisia; 4 Department of Exercise and Health Sciences, University of Taipei, Taiwan; 5 High Performance Unit, Chinese Taipei Football Association, New Taipei City, Taiwan; 6 Exercise and Health Promotion Association, New Taipei City, Taiwan; 7 Naufar, Wellness and Recovery Center, Doha, Qatar

**Keywords:** Biological maturation, Physical metrics, Technical performance, Youth soccer, Soccer training, GPS devices

## Abstract

We examined the impact of biological maturity status ((pre-, circa- and post-peak height velocity (PHV)) on time-motion, physiological and technical responses of under-15 soccer players during different small-sided game (SSG) formats performed on different pitch sizes. Thirty-six players (n = 12 per biological maturity group) performed 3- and 4-a-side SSGs on regular (36 × 27 m, 40 × 30 m, respectively) and large (40 × 29 m, 44 × 33 m, respectively) pitches. Total distance (TD), peak velocity, accelerations (Accl) > 2.5 m · s^−2^, decelerations (Dec) > 2.5 m · s^−2^, distances covered at different speeds (0 to < 7.0, 7.0 to < 14.0, 14.0 to < 18.0, and ≥ 18.0 km · h^−1^), peak heart rate (HRpeak), HRmean (expressed as percentage of HRmax), rating of perceived exertion (RPE), post-SSG blood lactate concentration (BLa), and technical actions were recorded. There was a biological maturity effect on RPE, HRmean (%HRmax), percentage of successful passes (%SP), and interceptions, with pre-PHV players showing higher RPE and interception scores in large SSG formats (p < 0.05), and post-PHV players demonstrating higher HRmean (%HRmax) and %SP on regular and large 3-a-side and 4-a-side pitches (p < 0.05). Across all biological maturity groups, 4-a-side resulted in higher TD, distances covered at different speeds and ball-loss scores, and lower Accl, Dec, BLa, %SP, and duel scores than 3-a-side SSGs (p < 0.05) for both pitch sizes. On large pitches, 3-a-side SSGs resulted in higher ball possession, interception, and RPE scores (p < 0.05), while regular pitch sizes favoured higher Accl and Dec in both 3- and 4-a-side SSGs (p < 0.05). Large SSG formats generally outperformed regular formats in time-motion metrics. Our findings underscore the importance of considering both biological maturity and game format when designing training sessions for youth soccer players.

## INTRODUCTION

Small-sided games (SSGs) have become a key aspect of soccer training, replicating match conditions in a focused, intense environment [1, 2]. SSGs effectively enhance technical, tactical, and physical skills, especially in youth soccer, supporting balanced skill acquisition, game understanding, and physical development [3, 4].

Research has examined SSG characteristics such as pitch alterations [4] and team size [5] or rule changes [6]. The 3-a-side and 4-a-side formats are notable for their impact on player development with common characteristics and some slight differences of outcome [7]. For instance, the 3-a-side format increases physical demands due to more relative space, encouraging high-intensity actions, one-on-one situations, and rapid transitions, enhancing technical skills and decision-making [3]. The 4-a-side format promotes greater tactical involvement and aerobic conditioning when the relative area per player is increased, resulting in more extensive player distribution on the pitch [7]. The characteristics of different game formats highlight the need to adjust pitch size (small, regular, large) to optimize players’ time-motion metrics and responses. Previous research showed that reducing the playing area-per-player increases technical demands, while enlarging it boosts physical demands and surface coverage [2]. Lemes et al. [8], focusing on the 3-a-side format across age groups, reported that pitch size (regular vs. large) may not impact the time-motion or physiological responses in all age categories, but differences between age categories were observed. However, Santos et al. [4] reported that larger fields led to more distance covered and increased high-intensity running in young age groups compared to older players.

Examining the interaction between game format and pitch size during SSGs offers valuable insights, especially given the significant physical and biological changes during adolescence [9]. Adolescence brings changes in body size and maturation, making talent identification complex [10]. This biological maturation, involving changes in height, weight, muscle mass, and cardiovascular development, occurs asynchronously across players in the same age group [11]. Early maturers tend to be bigger and taller [12, 13], giving them physical advantages in motor abilities [14] and the likelihood to be selected [15]. These advantages diminish by the end of adolescence, meaning that the early elimination of players delayed in biological maturity, relative to age-matched peers, can have long-term negative effects [9, 15]. Previous studies have reported that biological maturity significantly influences physical performance in soccer, particularly in metrics such as peak running speed and high-speed running [16, 17]. For instance, early-maturing male players often outperform their later-maturing peers in peak running speed and the frequency of high-speed running actions during matches and training [16, 17]. Buchheit and Mendez-Villanueva [16] found that advanced biological maturity status correlates with greater match running performance, including higher peak running speeds in under-15 (U15) soccer players. Similarly, Gundersen et al. [17] demonstrated that early-maturing players engage in more high-speed running and maintain higher peak running speeds, reflecting the physical advantages conferred by advanced biological maturity status. These findings highlight the edge that biologically advanced players hold, which can influence selection processes and development pathways in youth soccer. Da Costa et al. [9] found that in male U15 players, technical actions during SSGs were negatively associated with biological maturity, body size, and motor performance, suggesting that factors beyond anthropometrics, such as technical skills and tactical knowledge, influence motor performance. Similarly, Nunes et al. [18] concluded that male U15 players in mid-puberty may experience growth-related changes that affect their ability to execute technical skills, further highlighting the influence of maturation on technical performance [11]. Bio-banding, where players are grouped by biological maturity status rather than chronological age, aims to reduce inter-individual variation in maturity within groups, potentially creating a more balanced training environment for players with different maturity statuses. This approach can help mitigate disadvantages for late maturers by ensuring they compete with peers with similar levels of biological maturity status [19, 20].

The pitch size and number of players of SSGs are the most studied variables in youth soccer players, but, surprisingly, most of the studies only assess one game format or use a different methodology that does not allow comparisons to be made (e.g., different chronological age categories playing on the same pitch size [4]). Some studies have investigated the relationship between different SSGs (i.e., pitch size, team size) and their effect in different age categories. However, to the best of our knowledge, no study has investigated the SSG conditions with players of the same chronological age but different biological maturity status. Understanding how different SSG formats and pitch sizes interact with players’ biological maturity (i.e., peak height velocity (PHV)), and their outcome on the players’ time-motion, physiological and technical responses will provide insights that can enhance training regimens, ensure fair competition, and optimize player development. Thus, we aimed to examine the impact of biological maturity status (i.e., pre-, circa- and post-PHV) on the technical, time-motion and physiological responses of young male soccer players (i.e., U15) during different SSG formats (i.e., 3- and 4-a-side) performed on different pitch sizes (i.e., regular and large pitch sizes).

## MATERIALS AND METHODS

### Participants

Prior to the recruitment process, a sample size estimation was performed using the G*Power software (version 3.1.9.4, University of Kiel, Kiel, Germany). The study design involved an ANOVA test with repeated measures and within-between interaction. Effect sizes for the estimation were derived from tabled data in previous research [21]. The analysis determined that 10 participants per group were required to detect differences, with a Type I error rate of 0.05 and a Type II error rate of 0.20 (statistical power = 80%, actual power = 80.9%). Thus, 71 elite male youth soccer players competing in the U15 Tunisian National League volunteered to participate in this study. The participants were assessed for maturity status using the Mirwald method [22], a cost-effective and noninvasive approach relying on anthropometric measurements to estimate years from peak height velocity (PHV). The maturity offset was calculated as follows: maturity offset = -9.236 + (0.0002708 × leg length × sitting height) + (-0.001663 × age × leg length) + (0.007216 × age × sitting height) + (0.02292 × body mass/body height ratio). This offset facilitated the categorization of individuals into three distinct groups based on PHV offset: pre-PHV (-3 to > -1 years), circa-PHV (-1 to +1 year), and post-PHV (> 1 to +3 years). The maturity status assessment resulted in 21 pre-, 31 circa- and 19 post-PHV players. Each age category was required to include four defenders, four midfielders and four forwards. To facilitate the selection process, the four players who obtained the highest scores in each playing position on a specific soccer skill test, previously administered by the coach, were chosen to complete participation in the study [23].

To be eligible to participate in the study, individuals had to meet the following inclusion criteria: (a) being free from (i) severe musculoskeletal injuries for at least one year and (ii) mild to moderate injury for the month preceding the study [24]; (b) a minimum of five years of soccer experience with consistent engagement in the club’s training routines. Participant characteristics per biological maturity status are illustrated in Table 1. This study received institutional ethics approval (approval number: 004/2020; date of approval: February 11, 2020) and was conducted in accordance with the Declaration of Helsinki. All participants provided written informed consent from their parents or legal guardians before beginning the trial.

**TABLE 1:** Participants’ physical characteristics (mean ± SD) (n = 36).

	Age (years)	Height (cm)	Body mass (kg)	BMI (kg · m^-²^)	LL (cm)	SH (cm)	PHV (years)	APHV (years)
Pre-PHV (n = 12)	14.7 ± 0.4	167.4 ± 8.5	52.9 ± 6.2	19.02 ± 0.9	86.1 ± 2.1	81.3 ± 3.1	–2.1 ± 0.4	12.6 ± 0.3
Circa-PHV (n = 12)	14.4 ± 0.9	164.8 ± 7.6	51.8 ± 6.7	19.32 ± 2.4	83.9 ± 3.6	80.9 ± 3.7	−0.6 ± 0.3	13.8 ± 0.2
Post-PHV (n = 12)	14.2 ± 0.7	162.4 ± 6.2	50.3 ± 5.8	19.22 ± 1.7	82.6 ± 2.4	79.8 ± 2.5	1.2 ± 0.2	15.4 ± 0.2

Note: SD: standard deviation; BMI: body mass index; LL: leg length; SH: sitting height; PHV: peak height velocity; APHV: age of peak height velocity.

### Experimental Design

**FIG. 1:**
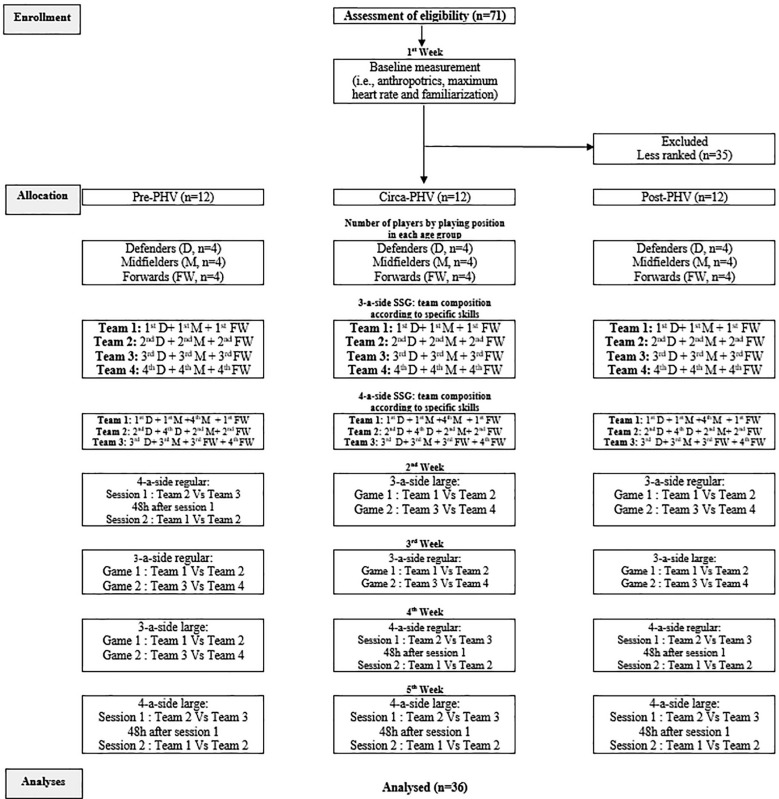
Team composition procedures within each group.

The current study adopted a cross-sectional design to examine the impact of biological maturity status (i.e., pre-, circa- and post-PHV) on the time-motion, physiological and technical responses of young male soccer players during different SSG formats (i.e., 3- and 4-a-side) performed on different pitch sizes (i.e., regular and large). The study was conducted during the soccer in-season period (February 2023, while the seasons started in September 2022) and lasted 5 weeks. The first week was devoted to assessing the anthropometric parameters (body mass was measured to the nearest 0.1 kg using a digital scale (OHAUS, Florhman Park, NJ, USA), body height, leg length, and sitting height were measured to the nearest 0.01 m, and body mass index (BMI) was determined (kg · m-^2^)). Maximal heart rate (HRmax) was measured during the Yo-Yo Intermittent Recovery Test Level 1 (YYIRT1) as described by Bangsbo et al. [25]. During that week participants became familiar with wearing the global positioning system (GPS) devices and heart rate monitors (Polar Team Pro, Kempele, Finland), as well as practising each of the soccer SSG formats (i.e., 3- and 4-a-side on regular, and 3- and 4-a-side on large pitches). The remaining four weeks were used for the experimental assessment administering the SSG formats (one format per week). Based on specific soccer skills, each player was ranked by the coach with respect to his abilities in passing, close ball control, shooting, and game sense using a 5-point Likert scale (1 = “outstanding,” to 5 = “below average”) [23]. Within each biological maturity group, teams were arranged according to the ranking in the specific soccer skill results [23] and game position (Figure 1). For example, in the first 3-a-side confrontation, team 1 consisted of the best forward, best midfielder and best defender, while team 2 consisted of the second-best forward, second-best midfielder and second-best defender. This counterbalanced procedure was adopted to allow similar technical performance conditions within teams [9]. The procedures regarding the teams’ composition are described in Figure 1. The order of SSG formats was established randomly using a random generator (Figure 1). To minimize the influence of fatigue, all SSG sessions were conducted with a minimum interval of 48 hours after the latest weekly match or before the next game or intensive training session. These assessments were carried out under standardized conditions to ensure consistency and reliability across measurements.

### Procedures

#### Small-Sided Games

To ensure the validity and reliability of the findings, several measures were taken to control for potential confounding variables. All SSG sessions were conducted under similar environmental conditions. Temperatures ranged between 15°C and 21°C, with humidity levels ranging from 70% to 78%. These conditions were consistent to minimize their impact on time-motion and physiological responses. The games were played at the same time of the day, at the beginning of the training session (16 to 18 h), and conducted on synthetic grass soccer surface. As mentioned above, participants were engaged in structured 3-, 4-a-side regular and 3-, 4-a-side large pitch formats, with a goalkeeper in all formats. All SSG formats comprised two periods (halves) lasting 4 min each, interspersed with one minute of passive recovery. The two formats, 3-a-side and 4-a-side SSGs, were performed in two experimental conditions: regular and large pitch sizes. The regular pitch size for the 3-a-side format was 36 × 27 m [8], while the regular pitch size for the 4-a-side format was 40 × 30 m [26]. The large pitch sizes corresponded to an approximate 10% increase over the regular sizes [8], resulting in dimensions of 40 × 29 m for 3-a-side and 44 × 33 m for 4-a-side. These pitch sizes were chosen to meet the spatial and physical demands appropriate for U15 players. [8, 26]. Adapted 6 × 2 m goals were used [8], and players were instructed to strive for victory by scoring more goals than the opposing team. Verbal encouragement was provided, but no technical or tactical instructions were allowed. The participant performed the same warm-up routine each week, lasting 20 minutes and including a combination of cardiovascular exercises, agility drills, and dynamic stretches to enhance flexibility and coordination. Time-motion physical performance, technical actions, heart rate (HR), blood lactate concentration (BLa) and rating of perceived exertion (RPE) were measured. Players wore the same GPS device and HR monitor across all data collection sessions to ensure data uniformity and accuracy. Two coaches circled the pitch, promptly supplying new balls for uninterrupted play.

### Physiological responses

#### Heart rate

Exercising heart rate was recorded at 1 Hz using HR monitors (Polar, FS1, Kempele, Finland). The variables in this investigation were HRpeak, representing the highest recorded value during the two bouts of SSGs, and HRmean, representing the average of HR collected from the two bouts of SSG exercises. To standardize the intensity of exercise across different groups, HR values were expressed as a percentage of the HRmax.

### Lactate concentration

Blood lactate concentrations were assessed following a 3-minute period of post-SSG passive recovery using a Lactate Pro analyser (Arkray Inc., Kyoto, Japan) [27].

### Rating of perceived exertion

After every SSG, each player rated their effort using Borg’s 10-point Likert scale, where ‘1’ (in arbitrary units AU) meant the least effort and ‘10’ meant the highest [28].

### Time-motion physical responses

GPS units (SPI ProX; GPSports, Canberra, Australia) were used to record time-motion data of each SSG bout, capturing data directly at 10 Hz. The efficacy of 10-Hz GPS technology has been previously established as dependable and accurate in evaluating movement profiles in team sports [29]. The analysed variables included the total distance covered (TD), peak velocity (Vpeak) (m · s^−1^), number of acceleration (Accl) > 2.5 m · s^−2^ and deceleration (Dec) > 2.5 m · s^−2^, and the distances covered (in m) at specific speed zones as categorised previously [30] (i.e., stationary/walking (zone 1): 0 to < 7.0 km · h^−1^, low-intensity running (zone 2): 7.0 to < 14.0 km · h^−1^, medium-intensity running (zone 3): 14.0 to < 18.0 km · h^−1^, and high-intensity running (zone 4): ≥ 18.0 km · h^−1^. Data extraction was performed using the Team AMS R1 2016 software. Players consistently wore the same GPS device across all data collection sessions, for data uniformity and accuracy.

### Video technical analysis

The SSGs were recorded using a video camcorder (Sony Handycam DVD 850) positioned 3 m above and 10 m from the side line and perpendicular to the field of play. Technical actions during SSGs (i.e., total number of individual duels, percentage of individual successful passes (%SP), total number of individual ball losses (BL), total number of individual headers (HB), number of individual interceptions and total number of individual ball possessions (BP)) were recorded using a hand notation system [31, 32]. To assess the reliability of this analysis system, the same investigator analysed the SSG videos twice. The intra-observer agreement was evaluated using the kappa coefficient [33]. Kappa values for the analysed variables ranged from 0.87 to 0.96, demonstrating a high level of reproducibility in the analysis system.

### Statistical analysis

Descriptive data are presented as means ± standard deviation (SD). Prior to performing inferential statistics, the Shapiro–Wilk test was used to assess whether the variables followed a normal distribution, and Levene’s test was used to verify the homogeneity of variances. Variables that did not exhibit normality were transformed using a logarithmic equation. A three-way repeated measures analysis of variance (ANOVA) was applied to test the effects of biological maturity status (i.e., pre-, circa-, and post-PHV), game format (3-a-side and 4-a-side), and pitch size (regular and large) on the dependent variables during SSGs. When significant main effects or interactions were found, Bonferroni post hoc analyses were performed to identify specific pairwise differences. Effect sizes (ES) were calculated to estimate the strength of significant findings, with partial eta squared values converted to Cohen’s d [34]. The interpretation of effect size values was as follows: < 0.20: trivial effect, 0.20 to < 0.50: small effect, 0.50 to < 0.80: intermediate effect, and ≥ 0.80: large effect [31]. All statistical analyses were conducted using SPSS software v.26 for Windows (IBM Corp, Armonk, NY, USA), and the significance level was set at p < 0.05.

## RESULTS

Absolute values and ANOVA analysis results are displayed in Tables 2, 3, 4 and 5.

**TABLE 2:** Total distance covered, peak velocity, acceleration and deceleration responses during different soccer small-sided game formats in pre-, circa- and post-PHV groups (n = 36).

Variables	Groups	Formats						3-a-side vs. 4-a-side	ANOVA

		3-a-side			4-a-side				

		Regular	Large	Within comparison p(d)	Regular	Large	Within comparison p(d)		
TD (m)	Pre-PHV	963.9 ± 41.7	980.3 ± 35.9¶	< 0.0001 (0.42)	991.4 ± 40.0	1016.9± 34.9¶	> 0.0001 (0.68)	R: > 0.0001 (0.674)† L: > 0.0001 (1.036)†	Size: F_1.33_ = 108.646; p < 0.0001; ES = 1.753 Format: F_1.33_ = 915.660; p < 0.0001; ES = 5.112 Maturity: F_2.33_ = 0.193; p = 0.826; ES = 0 Size × Maturity: F_2.33_ = 6.659; p = 0.004; ES = 0.561 Size × Format: F_1.33_ = 119.276; p < 0.0001; ES = 1.838 Format × Maturity: F_2.33_ = 31.147; p < 0.0001; ES = 1.294 Format × Size × Maturity: F_2.33_ = 23.212; p < 0.0001; ES = 1.111

	Circa-PHV	964.3 ± 39.5	971.5 ± 41.2	NS	990.8 ± 40.3	1017.9 ± 40.1 ¶	< 0.0001 (0.676)	R: < 0.0001 (0.666) † L: < 0.0001 (1.14) †	

	Post-PHV	957.9 ± 40.2	962.8 ± 32.2 ¶	0.001 (0.133)	991.8 ± 39.0	1023.2 ± 40.0 ¶	< 0.0001 (0.793)	R: < 0.0001 (0.856) † L: < 0.0001 (1.664) †	

Vpeak (m · s^-1^)	Pre-PHV	17.5 ± 1.1	17.7 ± 1.0	NS	17.8 ± 1.3	18.2 ± 1.2	NS	R: NS L: NS	Size: F_1.33_ = 6.836; p = 0.013; ES = 0.408 Format: F_1.33_ = 19.075; p < 0.0001; ES = 0.719 Maturity: F_2.33_ = 0.051; p = 0.950; ES = 0 Size × Maturity F_2.33_ = 0.191; p = 0.827; ES = 0 Size × Format: F_1.33_ = 0.534; p <0.470; ES = 0 Format × Maturity: F_2.33_ = 0.860; p = 0.432; ES = 0 Format × Size × Maturity: F_2.33_ = 0.615; p = 0.547; ES = 0

	Circa-PHV	18.2 ± 1.2	17.8 ± 0.8	NS	18.0 ± 0.7	18.4 ± 0.8	NS	R: NS L: NS	

	Post-PHV	17.1 ± 1.0	17.4 ± 1.4	NS	18.2 ± 1.1	18.8 ± 1.4 ¶	0.040 (0.667)	R: NS L: NS	

Accl > 2.5 m · s^-2^ (n)	Pre-PHV	15.3 ± 0.8#	16.2 ± 0.8	0.002 (1.031)	14.3 ± 0.8#	15.2 ± 1.0	0.010 (0.909)	R: 0.010 (1.205)‡ L: 0.013 (1.129)‡	Size: F_1.33_ = 59.779; p < 0.0001; ES = 1.296 Format: F_1.33_ = 64.896; p < 0.0001; ES = 1.351 Maturity: F_2.33_ = 0.178; p = 0.838; ES = 0 Size × Maturity: F_2.33_ = 0.252; p = 0.779; ES = 0 Size × Format: F_1.33_ = 1.266; p = 0.269; ES = 0.087 Format × Maturity: F_2.33_ = 0.368; p = 0.695; ES = 0 Format × Size × Maturity F_2.33_ = 0.387; p = 0.682; ES = 0

	Circa-PHV	15.6 ± 0.9#	16.3 ± 0.8	0.005 (0.806)	14.1 ± 0.7#	14.1 ± 0.8	< 0.0001 (0)	R: 0.037 (1.953) ‡ L: < 0.0001 (2.747)‡	

	Post-PHV	15.4 ± 0.8#	16.3 ± 1.0	0.010 (1.52)	14.3 ± 1.0#	15.5 ± 0.9	0.003 (1.152)	R: 0.005 (1.305)‡ L: < 0.0001 (0.891)‡	

Dec > 2.5 m · s^-2^ (n)	Pre-PHV	17.7 ± 0.8#	19.1 ± 0.9	0.013 (1.687)	16.2 ± 0.9#	18.2 ± 0.8	0.001 (2.256)	R: 0.002 (1.728) ‡ L: < 0.0001 (1.069) ‡	Size: F_1.33_ = 43.458; p < 0.0001; ES = 1.101 Format: F_1.33_ = 84.656; p < 0.0001; ES = 1.546 Maturity: F_2.33_ = 0.171; p = 0.843; ES = 0 Size × Maturity: F_2.33_ = 0.129; p = 0.879; ES = 0 Size × Format: F_1.33_ = 2.152; p = 0.152; ES = 0.181 Format × Maturity: F_2.33_ = 0.347; p = 0.709; ES = 0 Format × Size × Maturity: F_2.33_ = 0.209; p = 0.813; ES = 0

	Circa-PHV	17.8 ± 1.0#	19.2 ± 0.8	0.002 (2.12)	16.4 ± 1.0#	18.0 ± 1.0	0.003 (1.566)	R: 0.002 (0.812) ‡ L: < 0.0001 (1.321) ‡	

	Post-PHV	17.6 ± 0.9#	19.3 ± 1.2	0.023 (1.559)	16.3 ± 1.4#	18.4 ± 1.2	0.005 (1.638)	R: < 0.0001 (1.084) ‡ L: < 0.0001 (0.702) ‡	

Note: Values are given as means ± SD; TD: total distance covered; Vpeak: peak velocity; Accl: acceleration; Dec: Deceleration; ES: effect size; †: Significantly different from 3-a-side SSGs; NS: not significant. ‡: Significantly different from 4-a-side SSGs; #: Significantly different from large pitch size; ¶: Significantly different from regular pitch size; R: regular; L: large.

**TABLE 3:** Distances covered in different speed zones during different soccer small-sided game formats in pre-, circa- and post-PHV groups (n = 36).

Variables	Groups	Formats						3-a-side vs. 4-a-side	ANOVA

		3-a-side			4-a-side				

		Regular	Large	Within comparison p(d)	Regular	Large	Within comparison p(d)		
Zone 1 (m)#	Pre-PHV	343.2 ± 36.8	351.4 ± 32.9¶	< 0.0001 (0.25)	350.1 ± 36.8	359.9 ± 32.5¶	< 0.0001 (0.25)	R: < 0.0001 (0.25)† L: < 0.0001 (0.26)†	Size: F_1.33_ = 49.039; p < 0.0001; ES = 1.176 Format: F_1.33_ = 405.585; p < 0.0001; ES = 3.399 Maturity: F_2.33_ = 0.01; p = 0.99; ES = 0 Size × Maturity: F_2.33_ = 1.345; p = 0.274; ES = 0.138 Size × Format: F_1.33_ = 39.924; p < 0.0001; ES = 1.054 Format × Maturity: F_2.33_ = 7.757; p = 0.002; ES = 0.604 Format × Size × Maturity: F_2.33_ = 4.444; p = 0.02; ES = 0.437

	Circa-PHV	342.8 ± 29.7	345.3 ± 29.3	NS	350.3 ± 30.0	359.0 ± 29.7¶	< 0.0001 (0.33)	R: < 0.0001 (0.33)† L: < 0.0001 (0.667)†	

	Post-PHV	340.4 ± 23.3	344.2 ± 23.5¶	0.0365 (0)	350.4 ± 23.4	359.4 ± 23.9¶	< 0.0001 (0.5)	R: < 0.0001 (0.5)† L: < 0.0001 (1)†	

Zone 2 (m)#	Pre-PHV	427.1 ± 13.4	430.3 ± 13.8¶	0.026 (0)	436.4 ± 13.9	443.3 ± 13.9¶	< 0.0001 (1)	R: < 0.0001 (0)† L: < 0.0001 (1)†	Size: F_1.33_ = 210.434; p < 0.0001; ES = 2.446 Format: F_1.33_ = 502.163; p < 0.0001; ES = 3.784 Maturity: F_2.33_ = 0.001; p = 0.999; ES = 0 Size × Maturity: F_2.33_ = 4.834; p = 0.014; ES = 0.461 Size × Format: F_1.33_ = 38.2; p < 0.0001; ES = 1.031 Format × Maturity: F_2.33_ = 10.774; p < 0.0001; ES = 0.737 Format × Size × Maturity: F_2.33_ = 1.535; p = 0.230; ES = 0.172

	Circa-PHV	427.1 ± 14.1	428.9 ± 12.1	NS	436.1 ± 15.5	444.8 ± 15.2¶	< 0.0001 (1)	R: < 0.0001 (0)† L: < 0.0001 (1)†	

	Post-PHV	423.1 ± 12.0	427.3 ± 11.0¶	0.005 (1)	437.0 ± 11.0	448.7 ± 11.4¶	< 0.0001 (1)	R: < 0.0001 (1)† L: < 0.0001 (1)†	

Zone 3 (m)#	Pre-PHV	188.1 ± 13.9	192.6 ± 14.4	NS	198.5 ± 13.5	206.0 ± 13.4	NS	R: < 0.0001 (0.784)† L: 0.002 (1.177)†	Size: F_1.33_ = 0.926; p = 343; ES = 0 Format: F_1.33_ = 193.518; p < 0.0001; ES = 2.345 Maturity: F_2.33_ = 1.115; p = 0.340; ES = 0.079 Size × Maturity: F_2.33_ = 16.104; p < 0.0001; ES = 0.916 Size × Format: F_1.33_ = 36.738; p < 0.0001; ES = 1.010 Format × Maturity: F_2.33_ = 14.57; p < 0.0001; ES = 0.868 Format × Size × Maturity: F_2.33_ = 18.285; p < 0.0001; ES = 0.980

	Circa-PHV	188.5 ± 16.1	191.0 ± 16.2	NS	197.8 ± 15.4	206.3 ± 14.8	NS	R: < 0.0001 (0.667) L: 0.001 (1.33)†	

	Post-PHV	188.9 ± 15.6	165.7 ± 9.9	NS	197.3 ± 15.3	206.9 ± 16.8	NS	R: < 0.0001 (0.667) L: 0 < 0.0001 (3.92)†	

Zone 4 (m)#	Pre-PHV	5.7 ± 1.1	5.9 ± 0.8	NS	6.4 ± 1.1	7.8 ± 1.1¶	< 0.0001 (1.227)	R: 0.001 (0.798)† L: < 0.0001 (2.173)†	Size: F_1.33_ = 22.878; p < 0.0001; ES = 0.791 Format: F_1.33_ = 108.936; p < 0.0001; ES = 1.756 Maturity: F_2.33_ = 0.221; p = 0.803; ES = 0 Size × Maturity: F_2.33_ = 0.187; p = 0.830; ES = 0 Size × Format: F_1.33_ = 11.081; p = 0.002; ES = 0.537 Format × Maturity: F_2.33_ = 3.641; p = 0.037; ES = 0.383 Format × Size × Maturity: F_2.33_ = 0.163; p = 0.850; ES = 0

	Circa-PHV	5.9 ± 1.2	6.3 ± 0.1	NS	6.7 ± 1.1	7.8 ± 1.5¶	< 0.0001 (0.931)	R: 0.001 (1.273)† L: 0.001 (0.665)†	

	Post-PHV	5.5 ± 0.9	5.7 ± 0.7	NS	7.1 ± 1.01	8.2 ± 1.3¶	< 0.0001 (1)	R: < 0.0001 (1.687)† L: < 0.0001 (2.942)†	

Note: Values are given as means ± SD; #: transformed data log^10^; Stationary/walking (zone 1): 0 to < 7.0 km · h^-1^, low-intensity running (zone 2): 7.0 to < 14.0 km · h^-1^, medium-intensity running (zone 3): 14.0 to < 18.0 km · h^-1^, and high-intensity running (zone 4) > 18 km · h^-1^; ¶: Significantly different from regular pitch size; †: Significantly different from 3-a-side SSGs; for other abbreviations see Table 2.

**TABLE 4:** Physiological responses during different soccer small-sided game formats in pre-, circa- and post-PHV groups (n = 36).

Variables	Groups	Formats						3-a-side vs. 4-a-side	ANOVA

		3-a-side			4-a-side				

		Regular	Large	Within comparison p(d)	Regular	Large	Within comparison p(d)		
HRpeak (bpm)	Pre-PHV	186.3 ± 3.7	186.6 ± 2.9	-	187.7 ± 2.6	188.0 ± 2.4	-	-	

	Circa-PHV	186.3 ± 2.8	186.8 ± 2.3	-	186.9 ± 2.1	188.5 ± 1.9	-	-	

	Post-PHV	188.3 ± 4.0	188.7 ± 3.3	-	189.3 ± 3.0	189.4 ± 3.1	-	-	

HRmean (bpm)	Pre-PHV	154.9 ± 5.8	155.6 ± 4.9	-	155.7 ± 5.9	157.7 ± 3.8	-	-	

	Circa-PHV	156.6 ± 4.6	155.1 ± 4.8	-	157.8 ± 4.8	158.5 ± 3.9	-	-	

	Post-PHV	159.8 ± 3.5	160.6 ± 5.1	-	160.0 ± 2.6	159.8 ± 4.4	-	-	

HRpeak (%HRmax)	Pre-PHV	93.21 ± 1.77	93.3 ± 1.6¶	0.001 (0.071)	93.9 ± 1.3	94.0 ± 0.7¶	0.001 (0.126)	R: NS L: NS	Size: F_1.33_ = 45.937; p < 0.0001; ES = 1.133 Format: F_1.33_ = 0.970; p = 0.332; ES = 0 Maturity: F_2.33_ = 2.426; p = 0.104; ES = 0.281 Size × Maturity: F_2.33_ = 5.707; p = 0.007; ES = 0.500 Size × Format: F_1.33_ = 2.106; p = 0.156; ES = 0.178 Format × Maturity: F_2.33_ = 0.237; p = 0.790; ES = 0 Format × Size × Maturity: F_2.33_ = 2.218; p = 0.125; ES = 0.260

	Circa-PHV	92.90 ± 1.43	93.1 ± 1.0	NS	93.2 ± 1.5	94.0 ± 0.7¶	< 0.0001 (0.67)	R: NS L: NS	

	Post-PHV	93.9 ± 1.7	94.1 ± 1.3¶	0.013 (0.132)	94.43 ± 1.25	94.5 ± 1.20	NS	R: NS L: NS	

HRmean (%HRmax)	Pre-PHV	77.49 ± 2.88	77.82 ± 2.25	NS	77.87 ± 2.99	78.9 ± 1.8¶	0.043 (0.405)	R: NS L: NS	Size: F_1.33_ = 13.042; p = 0.001; ES = 0.586 Format: F_1.33_ = 0.219; p = 0.643; ES = 0 Maturity: F_2.33_ = 5.226; p = 0.011; ES = 0.484 Size × Maturity: F_2.33_ = 0.747; p = 0.482; ES = 0 Size × Format: F_1.33_ = 1.086; p = 0.305; ES = 0.049 Format × Maturity: F_2.33_ = 0.199; p = 0.820; ES = 0 Format × Size × Maturity: F_2.33_ = 1.881; p = 0.68; ES = 0.221

	Circa-PHV	78.10 ± 2.39	77.35 ± 2.32	NS	78.56 ± 2.49	79.05 ± 1.5¶	0.002 (0.239)	R: NS L: NS	

	Post-PHV	79.75 ± 1.68	80.12 ± 2.20	NS	79.83 ± 1.18	79.75 ± 1.9	NS	R: NS L: NS	

BLa (mmol · L^-1^)	Pre-PHV	4.62 ± 0.06‡	4.61 ± 0.05	NS	4.54 ± 0.06	4.53 ± 0.05	NS	R: < 0.0001 (1.33)‡ L: < 0.0001 (1.6)‡	Size: F_1.33_ = 0.220; p = 0.642; ES = 0 Format: F_1.33_ = 127.984; p < 0.0001; ES = 1.904 Maturity: F_2.33_ = 0.008; p = 0.991; ES = 0 Size × Maturity: F_2.33_ = 0.004; p = 0.996; ES = 0 Size × Format: F_1.33_ = 1.792; p = 0.190; ES = 0.150 Format × Maturity: F_2.33_ = 0.012; p = 0.988; ES = 0 Format × Size × Maturity: F_2.33_ = 0.136; p = 0.873; ES = 0

	Circa-PHV	4.62 ± 0.08‡	4.61 ± 0.11	NS	4.53 ± 0.09	4.53 ± 0.10	NS	R: < 0.0001 (1.057)‡ L: < 0.0001 (0.761)‡	

	Post-PHV	4.63 ± 0.13‡	4.61 ± 0.07	NS	4.53 ± 0.14	4.53 ± 0.08	NS	R: < 0.0001 (0.74)‡ L: < 0.0001 (1.064)‡	

RPE (AU)	Pre-PHV	6.6 ± 0.5	6.4 ± 0.7	NS	6.1 ± 0.8	6.3 ± 0.8	NS	R: NS L: NS	Size: F_1.33_ = 0.657; p = 0.424; ES = 0 Format: F_1.33_ = 21.251; p < 0.0001; ES = 0.761 Maturity: F_2.33_ = 4.969; p = 0.013; ES = 0.469 Size × Maturity: F_2.33_ = 0.212; p = 0.810; ES = 0 Size × Format: F_1.33_ = 0.024; p = 0.879; ES = 0 Format × Maturity: F_2.33_ = 0.515; p = 0.602; ES = 0 Format × Size × Maturity: F_2.33_ = 0.449; p = 0.642; ES = 0

	Circa-PHV	6.5 ± 0.5	6.4 ± 0.5	NS	6 ± 0.7	5.9 ± 0.7	NS	R: NS L: NS	

	Post-PHV	6.3 ± 0.9	6.2 ± 0.4‡	NS	5.8 ± 0.6	5.5 ± 0.5	NS	R: NS L: 0.014 (1.325)‡	

Note: Values are given as means ± SD; HRpeak: peak heart rate; bpm: beats per minute; HRmax: maximum heart rate; HRmean: mean heart rate; BLa: blood lactate; AU: arbitrary unit; ‡: Significantly different from 4-a-side SSGs; ¶: Significantly different from regular pitch size; for other abbreviations see Table 2.

**TABLE 5:** Technical action responses during different soccer small-sided game formats in pre-, circa- and post-PHV groups (n = 36).

Variables	Groups	Formats						3-a-side vs. 4-a-side	ANOVA

		3-a-side			4-a-side				

		Regular	Large	Within comparison p(d)	Within comparison p(d)	Regular	Large		
Number of duels	Pre-PHV	12.3 ± 1.6	12.6 ± 1.7	NS	11.3 ± 1.1	11.2 ± 1.0	NS	R: 0.001 (0.739)‡ L: < 0.0001 (1.016)‡	Size: F_1.33_ = 0.003; p = 0.959; ES = 0 Format: F_1.33_ = 86.806; p < 0.0001; ES = 1.566 Maturity: F_2.33_ = 1.511; p = 0.236; ES = 0.168 Size × Maturity: F_2.33_ = 0.459; p = 0.636; ES = 0 Size × Format: F_1.33_ = 3.930; p = 0.056; ES = 0.289 Format × Maturity: F_2.33_ = 0.202; p = 0.818; ES = 0 Format × Size × Maturity: F_2.33_ = 0.381; p = 0.686; ES = 0

	Circa-PHV	12.1 ± 1.2	12.7 ± 1.4	NS	11.3 ± 1.2	11.1 ± 1.1	NS	R: 0.004 (0.65)‡ L: < 0.0001 (1.257)‡	

	Post-PHV	13.0 ± 1.3	12.8 ± 1.4	NS	12.1 ± 1.3	11.6 ± 1.4	NS	R: 0.002 (0.695)‡ L: < 0.0001 (0.857)‡	

Percentage of successful passes#	Pre-PHV	63.3 ± 3.5	60.9 ± 3.4	NS	59.9 ± 3.5	58.7 ± 3.1	NS	R: < 0.0001 (1.5)‡ L: 0.048 (1)‡	Size: F_1.33_ = 3.09; p = 0.088; ES = 0.244 Format: F_1.33_ = 112.765; p < 0.0001; ES = 1.787 Maturity: F_2.33_ = 4.852; p = 0.014; ES = 0.463 Size × Maturity: F_2.33_ = 0.150; p = 0.861; ES = 0 Size × Format: F_1.33_ = 1.488; p = 0.231; ES = 0.118 Format × Maturity: F_2.33_ = 4.997; p = 0.013; ES = 0.471 Format × Size × Maturity: F_2.33_ = 2.522; p = 0.096; ES = 0.291

	Circa-PHV	64.1 ± 4.1	64.1 ± 4.9	NS	61.3 ± 4.2	58.4 ± 2.9	NS	R: < 0.0001 (1)‡ L: < 0.0001 (1.569)‡	

	Post-PHV	67.6 ± 5.3	67.4 ± 3.9	NS	61.1 ± 4.2	60.4 ± 4.9	NS	R: < 0.0001 (1.177)‡ L: < 0.0001 (1.961)‡	

Number of lost balls#	Pre-PHV	9.3 ± 1.2	9.9 ± 1.2	NS	10.1 ± 1.3†	10.8 ± 0.1	NS	R: 0.029 (0.8)† L: NS	Size: F_1.33_ = 1.513; p = 0.227; ES = 0.121 Format: F_1.33_ = 59.284; p < 0.0001; ES = 1.290 Maturity: F_2.33_ = 2.485; p = 0.099; ES = 0.287 Size × Maturity: F_2.33_ = 0.202; p = 0.818; ES = 0 Size × Format: F_1.33_ = 5.244; p = 0.029; ES = 0.348 Format × Maturity: F_2.33_ = 3.420; p = 0.045; ES = 0.367 Format × Size × Maturity: F_2.33_ = 1.0265; p = 0.370; ES = 0.038

	Circa-PHV	9.2 ± 1.8	9.3 ± 1.8	NS	10.0 ± 1.7†	10.9 ± 1.4	NS	R: 0.010 (0.532)† L: < 0.000 (1.199)†	

	Post-PHV	8.6 ± 1.5	8.3 ± 1.4	NS	9.9 ± 1.2†	10.5 ± 1.6	NS	R: < 0.000 (1.151)† L: < 0.0001 (1.534)†	

Number of header balls	Pre-PHV	1.2 ± 0.9	1.0 ± 0.1	NS	1.4 ± 0.7	1.5 ± 0.5	NS	R: NS L: 0.018 (0.653)†	Size: F_1.33_ = 0.052; p = 0.821; ES = 0 Format: F_1.33_ = 13.692; p = 0.001; ES = 0.602 Maturity: F_2.33_ = 0.365; p = 0.697; ES = 0 Size × Maturity: F_2.33_ = 0.07; p = 0.933; ES = 0 Size × Format: F_1.33_ = 0.029; p = 0.865; ES = 0 Format × Maturity: F_2.33_ = 0.029; p = 0.664; ES = 0 Format × Size × Maturity: F_2.33_ = 1.867; p = 0.170; ES = 0.219

	Circa-PHV	1.3 ± 1.1	1.3 ± 1.1	NS	1.6 ± 0.8	1.8 ± 0.6	NS	R: NS L: 0.018 (0.549)†	

	Post-PHV	1.3 ± 0.8	1.3 ± 1.1	NS	1.7 ± 0.8	1.5 ± 1.6	NS	R: NS L: NS	

Number of interceptions	Pre-PHV	10.2 ± 1.1	11.2 ± 1.3¶	0.027 (0.816)	10.3 ± 1.2	11.0 ± 1.1	NS	R: NS L: NS	Size: F_1.33_ = 12.948; p = 0.001; ES = 0.584 Format: F_1.33_ = 9.841; p = 0.004; ES = 0.502 Maturity: F_2.33_ = 7.419; p = 0.002; ES = 0.597 Size × Maturity: F_2.33_ = 0.166; p = 0.848; ES = 0 Size × Format: F_1.33_ = 1.585; p = 0.217; ES = 0.129 Format × Maturity: F_2.33_ = 3.379; p = 0.046; ES = 0.363 Format × Size × Maturity: F_2.33_ = 1.537; p = 0.230; ES = 0.173

	Circa-PHV	11.3 ± 1.5	11.8 ± 0.9	NS	10.6 ± 1.2	11.8 ± 0.7¶	0.007 (1.3)	R: 0.031 (0.504)‡ L: NS	

	Post-PHV	12.2 ± 1.3	12.6 ± 1.2	NS	11.1 ± 1.4	11.9 ± 0.9	NS	R: 0.001 (0.8)‡ L: 0.035 (0.618)‡	

Number of ball possessions	Pre-PHV	32.6 ± 2.2‡	33.0 ± 1.8	NS	31.7 ± 1.7	31.8 ± 1.6	NS	R: 0.019 (0.467)‡ L: < 0.000 (0.734)‡	Size: F_1.33_ = 0.861; p = 0.360; ES = 0 Format: F_1.33_ = 38.39; p < 0.0001; ES = 1.033 Maturity: F_2.33_ = 0.220; p = 0.804; ES = 0 Size × Maturity: F_2.33_ = 0; p = 1.00; ES = 0 Size × Format: F_1.33_ = 1.455; p = 0.236; ES = 0.114 Format × Maturity: F_2.33_ = 0.125; p = 0.883; ES = 0 Format × Size × Maturity: F_2.33_ = 0.058; p = 0.944; ES = 0

	Circa-PHV	32.7 ± 1.3	33.1 ± 1.2	NS	31.9 ± 1.6	32.0 ± 0.1	NS	R: NS L: 0.001 (1.019)‡	

	Post-PHV	32.8 ± 1.8‡	33.2 ± 0.9	NS	32.0 ± 0.1	32.2 ± 0.9	NS	R: 0.032 (0.577)‡ L: 0.002 (1.075)‡	

Note: Values are given as means ± SD; #: transformed data log^10^; †: Significantly different from 3-a-side SSGs; ‡: Significantly different from 4-a-side; ¶: Significantly different from regular pitch size; for other abbreviations see Table 2.

There was a biological maturity effect for RPE, HRmean (%HRmax), %SP and interception scores (Tables 4 and 5). Pre-PHV had higher RPE scores than post-PHV in large 4-a-side SSGs (p = 0.030; d = 1.325), and higher interception scores than post-PHV in both regular (p = 0.002; d = 1.633) and large (p = 0.018; d = 1.104) 3-a-side SSGs. However, post-PHV had higher HRmean (%HRmax) than circa-PHV in regular 4-a-side SSGs (p = 0.015; d = 0.652), and higher %SP scores than pre-PHV in large 3-a-side SSGs (p = 0.001; d = 2.0). No main effects for biological maturity were observed in the other assessed variables (Tables 2, 3, 4, and 5).

Within all biological maturity status groups, TD, distances covered in all speed zones and BL scores were higher in 4-a-side than 3-a-side SSGs in both regular and large sizes (all p < 0.05) (Tables 2, 3, and 5). The 3-a-side SSGs resulted in higher Accl, Dec, BLa, %SP, and duel scores than 4-a-side SSGs in both regular and large sizes (all p < 0.05) (Tables 4 and 5). On the large pitches, the 3-a-side SSGs induced larger BP (all age groups, p < 0.05), interception (post-PHV, p < 0.05) and RPE (post-PHV, p < 0.05) scores than the 4-a-side (Tables 4 and 5). Moreover, the 4-a-side SSGs elicited lower interception (circa- and post-PHV, p < 0.05) and BP (pre- and post-PHV, p < 0.05) scores than 3-a-side SSGs on the regular size pitches (Tables 4 and 5).

When players were pooled across biological maturity status, the large 4-a-side SSGS resulted in higher TD, speed zones 1, 2 and 4 (all age groups, p < 0.05), Vpeak (post-PHV, p < 0.05), HRpeak and HRmean (%HRmax) (pre- and circa-PHV, p < 0.05), and interception (circa-PHV, p < 0.05) scores than the regular 4-a-side SSGs (Tables 2, 3 and 5). Also, the large 3-a-side SSGs induced higher TD, speed zones 1 and 2, HRpeak (%HRmax) (pre- and post-PHV, p < 0.05), and interception (pre-PHV, p < 0.05) scores than regular 3-a-side SSGs (Tables 2, 3 and 5). Regular pitch size induced higher Accl and Dec scores in both 3- and 4-a-side SSGs within all groups (p < 0.05) (Table 2). No main effect for pitch size was identified in speed zone 3, RPE, BLa, %SP, BL, HB, duel, and BP scores (Tables 3, 4, and 5).

## DISCUSSION

We aimed to examine the impact of biological maturity status on the time-motion, physiological and technical responses of male adolescent soccer players (i.e., U15) during different SSG formats performed on differently sized pitches.

To our knowledge, this is the first study investigating the technical actions, time-motion performances, and physiological responses in different SSG formats and pitch sizes according to biological maturity status in young soccer players. Despite being of the same chronological age, post-PHV players showed higher HRmean and percentage of successful passes, and lower RPE and ball interceptions scores than their pre-PHV counterparts. These differences, showcasing the complex maturation process, may be linked to physical [35] and physiological [36] development. A highly developed cardiovascular system can increase exercise capacity and efficiency resulting from the increased maturation seen in post-PHV players [36]. This development might explain the observed higher HRmean values, as post-PHV players can sustain higher workloads more efficiently [36]. The lower RPE in post-PHV players could also reflect this enhanced cardiovascular efficiency, as they might perceive the same physical load as less strenuous than their less mature peers [36]. We observed a larger %SP and fewer interceptions among post-PHV players, which may suggest differences in gameplay style or physical capacity associated with maturation. However, further research is needed to examine how biological maturity status might influence decision-making abilities, spatial awareness, and tactical knowledge in young players [37]. Indeed, these cognitive abilities are essential in soccer, as players must constantly evaluate the game environment and make split-second decisions [37]. Physical and technical skills are also affected by physiological changes brought on by maturity [35]. Post-PHV players frequently possess increased muscular mass and strength, which enhances their capacity to execute precise and potent movements [35]. Additionally, their enhanced physical development can enable them to occupy better defensive positions, hence decreasing the numbers of interceptions made by opponents [35, 38]. The combination of physical strength, aerobic capacity, and technical skill contributes to the players’ overall effectiveness on the field [35]. These results underscore the importance of considering biological maturity status and chronological age when developing training programmes and strategies for youth soccer players. To optimise development opportunities and reduce injury risk, coaches should ideally customise training and competition experiences to the maturity level of their athletes [39]. For example, whereas pre- and circa-PHV players may prioritise improving physical capacity and foundational abilities, post-PHV players may benefit more from training programmes that concentrate more on refining technical skills and tactical awareness. Youth sports programmes must acknowledge and accommodate these variances to assist young athletes’ holistic development and create conditions that maximise their short- and long-term athletic potential.

The results of the 3-a-side and 4-a-side SSGs reveal that time-motion physiological and technical responses vary across biological maturity. In all biological maturity groups, TD and distances covered at all speed zones were higher in 4-a-side games, likely due to the greater space distribution and more movement possibilities across the pitch. This is consistent with the findings of Sarmento et al. [7], who found that larger-sided games typically result in more time-motion output due to shared player responsibilities. In contrast, the 3-a-side format resulted in higher Accl, Dec, BLa, and %SP, particularly benefiting technical and high-intensity actions due to fewer players, aligning with Clemente et al. [3], who reported that smaller-sided games increase individual involvement and intensity.

Post-PHV players displayed higher interception and RPE scores in the 3-a-side SSGs on larger pitches compared to the other biological maturity group players, indicating that more physically mature players seem to better take advantage from the added space and tactical complexity. The latter observations support Ford et al. [40], who emphasize that older, more mature player can handle higher tactical and physiological demands. The higher ball possession in 3-a-side SSGs across all biological maturity groups also reflects the increased technical involvement and decision-making opportunities due to the reduced number of players [31]. The lower interception rates in 4-a-side games for circa- and post-PHV players highlight how the larger format reduces the need for frequent defensive actions, potentially leading to less individual involvement in high-intensity challenges, a point supported by Dellal et al. [41], who observed that smaller-sided games engage players more frequently in defensive duels and interceptions.

Moreover, in terms of physiological demand, the larger 4-a-side SSGs elicited higher Vpeak, HRpeak, and HRmean, especially in pre- and circa-PHV players, suggesting that larger-sided formats on bigger pitches impose more aerobic and endurance demands, as shown by Clemente et al. [3], who observed that the increased space in larger-sided games favours aerobic conditioning. In contrast, the larger 3-a-side games induced higher HRpeak in both pre- and post-PHV groups, indicating that while fewer players are involved, the individual intensity is greater, particularly for younger and more mature players [7].

Finally, regular pitch sizes led to higher Accl and Dec scores in both formats, likely due to the increased need for rapid changes of direction and pace in a more confined space, which enhances agility and reactive capacity, particularly in developing youth players [42]. This is consistent with Dellal et al. [41], who noted that smaller pitch sizes demand greater agility and frequent high-intensity efforts, critical for the development of youth players’ movement skills and quick decision-making abilities. Overall, these findings underscore the importance of tailoring game formats based on biological maturity status and maturation status to optimize time-motion performances, and physiological and technical development in youth soccer players. Adapting pitch size and game formats can enhance training outcomes, supporting players’ physical activity, tactical awareness, and skill development. This study clarifies differences between 3- and 4-a-side games’ time-motion, physiological and technical responses.

We acknowledge several limitations in this study. First, the predictive equation used for estimating the age at peak height velocity (PHV) (the Mirwald equation) may overestimate PHV in early-maturing youth and underestimate it in late-maturing youth. In this regard, it is important to note that this equation estimates the age at PHV rather than true biological age, which may impact the classification accuracy of biological maturity status [43]. Moreover**,** the homogeneity of our sample, consisting of a single age group of male players from a specific competitive level, may limit the generalizability of our findings to other competitive levels or geographical contexts. In addition, as the study included only male players, we did not examine potential biological sex differences. Therefore, our results should not be generalized to female players, who may exhibit different patterns in biological maturity and physical performance. Future research should consider including female participants, competitive levels, and geographic contexts to bring more granularity to the applicability of these findings.

## CONCLUSIONS

In conclusion, our study highlights the significant impact of biological maturity status on the time-motion, physiological, and technical responses of U15 male soccer players during SSGs. Post-PHV players exhibited a higher HRmean percentage of successful passes, and lower RPE, likely due to advanced physical and physiological development. Additionally, their superior decision-making and spatial awareness resulted in fewer ball interceptions. Differences in game formats, with higher intensity in 3-a-side and greater time-motion output in 4-a-side SSGs, underscore the importance of adapting training to players’ maturation levels. Coaches should consider biological maturity status when designing training programmes to enhance technical, tactical, and physical development, optimizing the long-term growth of youth players.

## Data Availability

Data are available from the first author upon reasonable request.
